# Comparative Laboratory Tests of Thermal Conductivity of Road Materials Using Two Measurement Methods

**DOI:** 10.3390/ma18091970

**Published:** 2025-04-26

**Authors:** Jarosław Górszczyk, Konrad Malicki

**Affiliations:** Faculty of Civil Engineering, Cracow University of Technology, 31-155 Cracow, Poland; kmalicki@pk.edu.pl

**Keywords:** thermal conductivity, heat transfer, asphalt mixture, cement concrete, steady-state thermal conduction, transient heat conduction, heat flux, sustainable road pavement

## Abstract

The fundamental material parameter used in the thermal analysis of road pavement structures is the thermal conductivity. This parameter can be determined using various methods. The main objective of this paper is to compare and evaluate the thermal conductivity test results obtained using two different measurement methods. Thermal conductivity was determined using the steady-state and transient methods. The transient method is more cost-effective and faster but tends to produce a higher dispersion of results. In contrast, the steady-state method is more challenging to apply, particularly when testing large and heavy specimens of heterogeneous materials such as road pavement materials. For this reason, it is essential to assess the differences in results obtained by these two methods when applied to road materials. Two types of materials were tested in this study: an asphalt mixture and a cement concrete. The obtained results show statistically significant differences (α = 0.05), taking into account the two methods considered. The average difference can be estimated at 10% and 11% for asphalt mixtures and cement concretes, respectively. The obtained results are important for quantifying material parameters used in thermal and coupled thermal/structural analysis of pavement structures. This is particularly relevant in areas affected by urban heat islands and in road sections used as solar collectors.

## 1. Introduction

The development of transport infrastructure is a key element in the sustainable economic growth of society. At the same time, a significant rise in air temperature is observed all over the world [1]. This issue is a serious challenge for societies and scientists. In urban areas, temperatures are typically higher than in surrounding rural regions. Factors influencing the accumulation of heat in cities include, among others, densely located buildings and heavily developed and utilized road networks. On the one hand, heat accumulation can adversely affect the environment and human health by creating urban heat islands (UHIs) [1]. In addition, increased road surface temperature can accelerate the process of pavement rutting and aging [2]. On the other hand, road pavements can function as heat collectors, enabling the acquisition of renewable energy from the sun. In the conditions of global warming, it is therefore necessary to design roads that enable sustainable socio-economic development [3]. Marcobal et al. emphasized that energy-efficient paving technologies should be directed towards more sustainable pavement management practices [4].

Thermal factors are considered in the analysis of road pavement structures [5]. According to Shin and Kodide, temperature effects are incorporated, for example, in the American road pavement design method [6]. Temperature also affects the strength of the asphalt interlayer bonding [7,8]. The phenomenon of the heat transfer in a multilayer road pavement structure can be described by the Fourier–Kirchhoff equation [9]. The heat flux q (W·m^−2^) depends on the thermal conductivity λ (W·m^−1^·K^−1^). Thermal conductivity λ determines the material’s ability to conduct heat. Thermal conductivity of materials can be tested using the steady-state or transient method [10]. The use of standard procedures for testing the heat transfer of concrete using the steady-state method (with a constant heat flow) is difficult. The steady-state method is more expensive and time-consuming than the transient method. Standard steady-state procedures are typically used for testing materials with thermal conductivity below 1.0 W·m^−1^·K^−1^ [6]. For these reasons, procedures utilizing the transient method for determining the thermal conductivity of materials are being developed at the same time [11]. Based on both original observations and a literature review, the most important advantages and limitations of the steady-state and transient methods are summarized in Table 1 [10,11].

In tests of insulating materials with a layer thickness of 10 cm, a high level of agreement with the results obtained using the steady-state method was observed. However, for thinner layers, significant differences may occur between the results obtained by the two methods [11]. For soil and cement mixtures the differences can be up to 20% [12]. Therefore, the question arises about the differences in the test results of the two methods for cement concretes and asphalt mixtures used in road pavement structures.

Due to the heterogeneity of concretes, their thermal conductivity depends on many factors. These include the type of aggregate, type and amount of binder, porosity, and humidity. The thermal conductivity of road bitumen binder is usually in the range of 0.15–0.35 W·m^−1^·K^−1^ [13,14]. Mrawira and Luca investigated the effect of aggregate type, gradation, and compaction level on thermal properties of asphalt mixtures [15]. The thermal conductivity of the asphalt concrete ranged from 1.7 to 2.1 W·m^−1^·K^−1^. The type of aggregate was found to have the most significant effect on the thermal parameters. The compaction level had an effect, but it was not statistically significant [15]. However, according to Hassn et al., asphalt mixtures with high air voids content exhibit lower thermal conductivity [16]. Cote et al. investigated thermal conductivity of asphalt concrete [17]. The paper demonstrates that the bitumen increases thermal conductivity compared to unbound aggregates. It was shown that the bitumen creates thermal bridges between aggregate grains similarly to other types of binders such as cement [17]. Dawson et al. presented enhancing thermal properties of asphalt materials for heat storage and transfer applications [18]. For the limestone asphalt mixture, a thermal conductivity of 1.21 W·m^−1^·K^−1^ was obtained. Fully replacing limestone aggregates with quartzite increased the thermal conductivity to 2.47 W·m^−1^·K^−1^. However, modifying the composition may have a negative impact on some of the mechanical parameters of the asphalt mixture. Pasetto et al. investigated innovative pavement surfaces as UHI mitigation strategy [1]. The results for five binder and aggregate mixtures prepared for the wearing course were presented. A combined effect of the surface color and the thermal conductivity on the heat flow was observed. Thermal conductivity depended on the parameters of the tested materials. The values obtained ranged from about 1.0 to 3.0 W·m^−1^·K^−1^. It was reported that the use of the clear resin as a binder allowed the enhancement of the thermal response of the tested mixtures [1]. Xu et al. presented thermal characteristics and temperature distribution of asphalt mixture specimens containing residues from municipal solid waste incineration (MSWIR) [19]. The mixtures with the addition of MSWIR showed thermal conductivity in the range of approximately 1.14 to 1.30 W·m^−1^·K^−1^. The transient plane heat source method was used. The voids in the MSWIR affected the temperature distribution within the asphalt mixture, inducing a thermal resistance effect. The increase in asphalt surface temperature caused by MSWIRs may exacerbate the urban heat island effect in the summer [19]. Kong et al. investigated the use of hybrid mineral filler (HMF) with high emissivity in asphalt mixture for cooling road pavements [2]. The asphalt mixture with HMF showed higher thermal conductivity than the control asphalt mixture. The combined effect of high emissivity and thermal conductivity led to up to 5.4 °C lower surface temperature of mixture with the highest HMF content compared to the control mixture [2].

Asadi et al. presented an overview of the thermal conductivity of cement concrete [10]. The test results of different types of cement concrete-based materials were collected. Thermal conductivity λ was shown to be in the range of about 0.1 to 3.8 W·m^−1^·K^−1^. The highest λ values were obtained for concretes with metal fibers. Thermal conductivity of concrete increases with increasing density [10]. Thermal conductivity of typical cement concretes usually ranges from 2.0 to 3.0 W·m^−1^·K^−1^ [6]. According to Neville, thermal conductivity of concrete depends on the type of aggregate used. For example, concrete made with granite aggregate has a thermal conductivity of approximately 2.6 W·m^−1^·K^−1^, whereas concrete with dolomite aggregate has a value of about 3.3 W·m^−1^·K^−1^ [20]. The thermal conductivity of cement mortar depends significantly on its composition and typically ranges from 0.9 to 1.5 W·m^−1^·K^−1^ [21,22]. Zhang et al. developed the mesoscale model for thermal conductivity of concrete [23]. It has been reported that the thermal conductivity of concrete increases with the increase in the volume content of coarse aggregate. The results of thermal conductivity tests of concrete with limestone coarse aggregate were presented. The results obtained by the guarded hot plate apparatus and transient plane source methods were compared. For the steady-state method, the average thermal conductivity value was 2.49 W·m^−1^·K^−1^, while for the transient method it was about 2.77 W·m^−1^·K^−1^ [23]. Benazzouk et al. analyzed thermal conductivity of cement composites containing rubber waste particles [24]. For the composite with 50% rubber particles addition, thermal conductivity of 0.47 W·m^−1^·K^−1^ was obtained.

Research is also carried out on the possibility of using asphalt pavements as solar collectors [25,26]. Abbas and Alhamdo presented experimental and numerical analysis of an asphalt solar collector (ASC) with a conductive asphalt mixture [26]. ASCs are made of tubes containing circulating fluids laid in pavement structure layers. Heat is transferred from the pavement to the fluid. Lowering road pavement temperature reduces the harmful effects of UHIs. It is concluded that the use of conductive mixtures significantly increases the thermal efficacy of an ASCs [26].

Numerical simulations are widely used in thermal analysis of pavement structures. Górszczyk and Grzybowska presented the use of finite element method (FEM) for thermal analysis of the asphalt pavement [27]. Pavement temperature distributions were presented for the analyzed variants. Wu et al. presented numerical simulation of temperature distribution in conductive asphalt solar collector taking into account pavement material parameters [28]. Graphite powders were used to improve the asphalt collector efficiency. A transient, two-dimensional finite element analysis was performed. Higher temperature gradient was observed at the depth of 10 cm because the heat conduction is accelerated by the addition of graphite. Han et al. proposed a multi-scale approach to modeling the thermal conductivity of aerogel-incorporated concrete [29]. The developed model takes into account various factors that impact the thermal conductivity of composite materials, such as the water–cement ratio, ingredients, age, and saturation level. The results showed the thermal conductivity in the range of approximately 0.25 to 3.0 W·m^−1^·K^−1^. She et al. presented numerical study on the effect of pore shapes on the thermal behavior of cellular concrete [30]. The FEM was adopted to simulate heat transfer through the microstructure. It was concluded that FEM can be an effective help for the material optimization.

Thermal conductivity is a fundamental material parameter used in thermal analysis of road pavements. It is determined in laboratory tests using one of two methods: the steady-state method or the transient method. For this reason, it is essential to evaluate the differences in the results obtained by these two methods. The primary objective of this research is to quantify differences in the test results, enabling the conscious use of the transient method in road material testing. The tests were conducted on two types of asphalt mixtures, described in detail in point number 2. Additionally, two cement concretes were comparatively tested [31,32]. Significant differences in thermal conductivity test results were confirmed and discussed.

## 2. Materials and Methods

### 2.1. Tested Asphalt Mixtures

**Figure 1:**
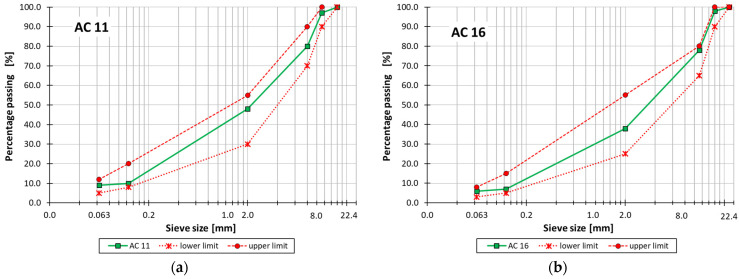
Gradation curves along with upper and lower limit curves for (**a**) AC 11 and (**b**) AC 16.

Two types of asphalt concrete (AC) with grain size up to 11 mm (AC 11) and up to 16 mm (AC 16) were used in the laboratory tests. The AC 11 mixture was intended for use as a wearing course, while the AC 16 mixture was intended for use as a binding course. Both asphalt mixtures were intended for the low traffic category. The gradation of mineral aggregate mixtures used for AC production is presented in Table 2, along with the limit curves shown in Figure 1.

The type of rock used in the coarse aggregate varied between mixtures. In AC 11, the coarse aggregate (grain size fractions 2/8 and 8/12) was made of dolomite rock. In AC 16, the coarse aggregate (grain size fractions 2/8 and 8/16) was made of limestone. The remaining mineral components of the mixtures were the same. Road bitumen with a penetration range of 50/70 and an adhesion agent were used in both formulations. The components of the asphalt mixtures are shown in Table 3.

**Figure 2:**
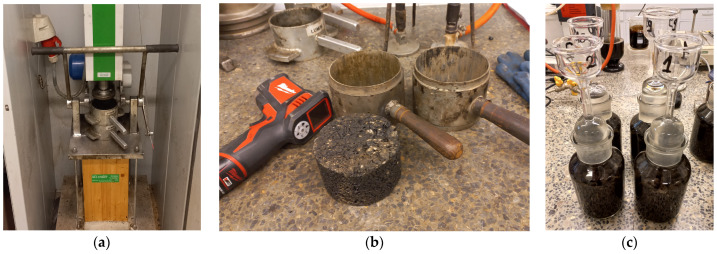
Preparation of specimens for evaluation of asphalt mixture parameters: (**a**) Marshall’s hammer; (**b**) Marshall’s molds and specimen; (**c**) pycnometers for determining density.

Marshall specimens were made to evaluate the properties of the asphalt mixtures. The Marshall hammer (Multiserw-Morek, Marcyporęba, Poland), molds, control specimen, and asphalt mixture in pycnometers are shown in Figure 2.

Density and voids were determined. The obtained results are summarized in Table 4. The mixtures used met the national technical requirements [33].

**Figure 3:**
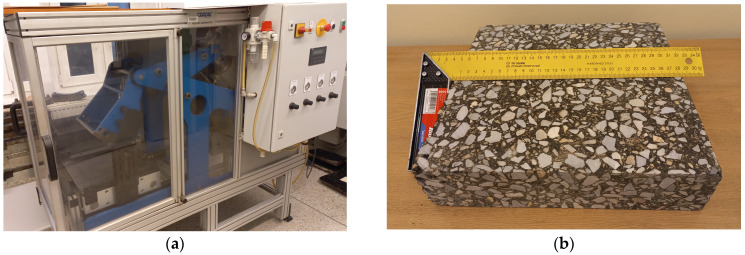
Preparation of plate specimens: (**a**) roller compactor; (**b**) specimen ready for testing.

In the next stage, plate specimens were prepared for thermal conductivity tests. The specimens were made in the laboratory using the roller compactor method, according to the standard [37]. The specimens were compacted at a temperature of 135 ± 5 °C. Plates with dimensions of 305 × 305 × 100 mm^3^ were made. In order to ensure flat side surfaces, the vertical edges of the specimens were cut off. Finally, plate specimens for thermal conductivity tests with dimensions of 285 × 285 × 100 mm^3^ were obtained. The laboratory roller compactor (Cooper Research Technology, Ripley, United Kingdom) and an example plate specimen are shown in Figure 3.

### 2.2. Tested Cement Concretes

**Figure 4:**
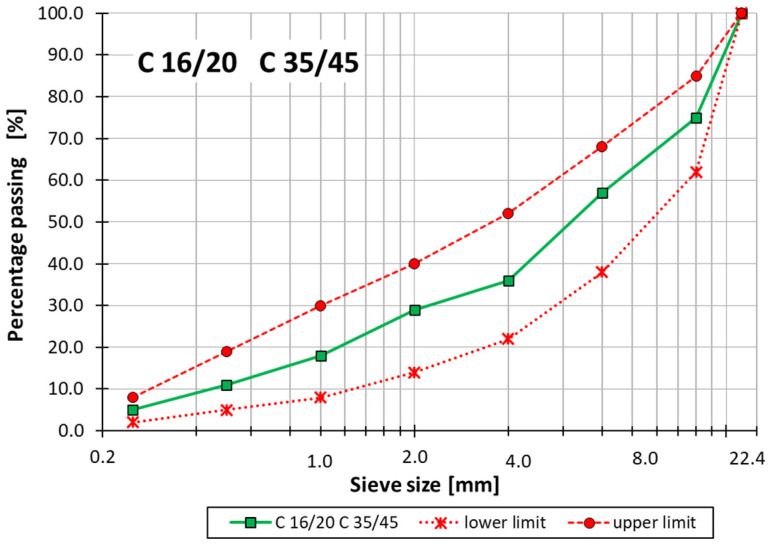
Gradation curve along with upper and lower limit curves of cement concretes (C 16/20 and C 35/45).

Two types of cement concretes for road pavements were used in the study. The concretes were designed in accordance with EN 206 [38] for a compressive strength class of 16/20 MPa (C 16/20) and for the strength class of 35/45 MPa (C 35/45). The higher strength concrete was designed for use as a top layer of the pavement, while the lower strength concrete was designed for use as a bottom layer. The same aggregate gradation was used in both mixtures. The mineral aggregate gradation is presented in Table 5, along with the limit curves shown in Figure 4.

The type of rock used in the coarse aggregate of the two tested concretes was varied. In C 16/20, the coarse aggregate (grain size fractions 2/8, 8/16 and 16/22 mm) was made of dolomite rock. In C 35/45, the coarse aggregate (grain size fractions 2/8, 8/16 and 16/22 mm) was made of granite rock. Low-alkaline Portland cements were used. In C 16/20, CEM III/A 42.5 N was used, while in C 35/45, it was CEM I 42.5 N. CEM III/A cement contained granulated blast furnace slag in the amount of 36%—65%. The cement parameters met the requirements of the EN 197-1 standard [39]. Tap water was used that met the requirements of the standard [39]. The w/c ratio for C 16/20 was 0.44, while for C 35/45 it was 0.42.

**Figure 5:**
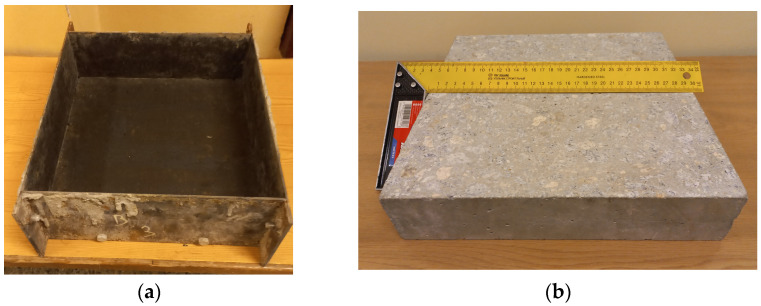
Preparation of plate specimens: (**a**) plate mold; (**b**) specimen ready for testing.

For thermal conductivity tests, plate specimens with dimensions of 300 × 300 × 100 mm^3^ were made. Concrete was made in a concrete plant and taken directly into molds. The mold for preparing specimens and the finished concrete specimen for thermal conductivity tests are shown in Figure 5.

### 2.3. Test Methods and Parameters

Thermal conductivity tests were of comparative nature. The steady-state and transient methods were used. The designations and numbers of the series of tested specimens are shown in Table 6.

Laboratory tests of steady-state heat transfer were carried out according to the EN 12667 [40] and ISO 8301 [41] standards. The Fox 802 plate device (Lasercomp, TA Instruments, New Castle, DE, USA) was used [42]. This device can operate within a temperature range of −10 °C to +65 °C. During the test, the temperature of the lower plate was set to +20 °C, while the upper plate was maintained at 0 °C. The heating and cooling of the plates were achieved using Peltier cells. The temperature measurement accuracy was ±1%, with a reproducibility of ±0.5%. The heat flow meter was equipped with optical encoders and stepper motors. These technical solutions enabled position control and measurement at all four plate corners [42]. In the laboratory procedure, additional external thermocouples were used, which enabled the tested range of thermal conductivity of concrete to be increased (up to 2.5 W·m^−1^·K^−1^). Thermal conductivity *λ* (W·m^−1^·K^−1^) was calculated according to Equation (1) [40].

(1) $$\lambda =\frac{\Phi \cdot d}{A\cdot (T_{1}-T_{2})}\;$$

where

λ—thermal conductivity ($W\cdot m^{-1}{\cdot K}^{-1}$);

*Φ*—average power supplied to the heating unit, heat flow ($J\cdot s^{-1}$);

*d*—specimen thickness (m);

*A*—metering area of the sample ${(m}^{2})$;

*T*_1_—average specimen hot side temperature (°C);

*T*_2_—average specimen cold side temperature (°C).

Transient method measures the response to a signal sent to produce heat flux in the specimen while steady-state method measures the response to a steady heat flux in the specimen. The transient method requires that the specimen first be in thermal equilibrium with its surroundings, then a short heating pulse is applied to the specimen. During this process, the instrument monitors the temperature change so that the thermal conductivity of the specimen material can be determined. Monitoring the specimen temperature as a function of time allows for additional calculation of the thermal diffusivity and volumetric heat capacity of the tested material. The relationship between thermal conductivity, thermal diffusivity and volumetric heat capacity is presented in Equation (2).

(2) $$\alpha =\frac{\lambda }{{vol\;c}_{p}}\;$$

where

***α***—thermal diffusivity ($m^{2}{\cdot s}^{-1}$);

λ—thermal conductivity ($W\cdot m^{-1}{\cdot K}^{-1}$);

${vol\;c}_{p}$—volumetric heat capacity ($J\cdot m^{-3}{\cdot K}^{-1}$).

**Figure 6:**
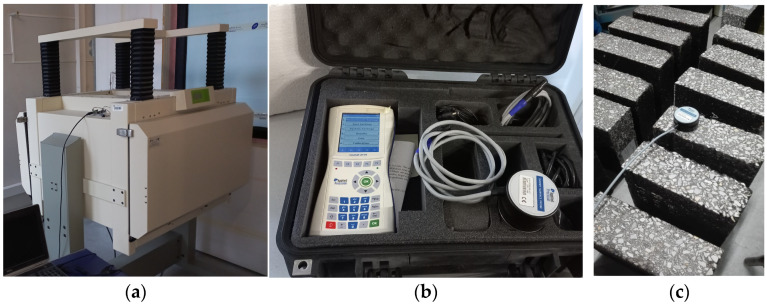
Thermal conductivity testing: (**a**) steady-state thermal conductivity measuring device Fox 802; (**b**) transient heat conduction apparatus Isomet 2114; (**c**) measurement of thermal conductivity of asphalt concrete using the transient heat conduction method.

The Isomet 2114 device (Applied Precision Ltd., Bratislava, Slovakia) was used for transient heat conduction tests. The device allows for measuring the thermal conductivity of hard materials using surface probes with a diameter of 6 cm. [43]. This enables measurement in the range of 0.04 to 6.0 W·m^−1^·K^−1^. The device operates in a temperature range of −15 to +50 °C. The measurement accuracy for thermal conductivity in the range of 0.7 to 6.0 W·m^−1^·K^−1^ is 10% of the reading value. The reproducibility of the measurement is 3% of the reading + 0.001 W·m^−1^·K^−1^ [43]. Laboratory tests using the transient method were carried out at a temperature of +24.5 ± 1 °C. The laboratory equipment and an example of measurement using the transient method are shown in Figure 6.

Thermal conductivity tests were carried out after the moisture content of the tested materials had stabilized. Moisture content was determined using the capacitive method based on the measurement of the dielectric constant of the material. The stabilized average moisture content of the tested asphalt mixture samples was 1.7%, and of the cement concrete 2.3%.

## 3. Results and Discussion

### 3.1. Test Results for Individual Materials

The steady-state method tests were performed separately for each specimen. The test results for the individual materials are presented in Table 7.

The results of the transient method tests are presented below. Due to the short measurement time of the transient method, the thermal conductivity tests were carried out for each specimen in three series, from which the mean values were calculated. The results of the thermal conductivity tests of asphalt mixtures are shown in Table 8 and Table 9. The results of the tests of cement concretes are shown in Table 10 and Table 11.

In the steady-state method tests, the mean thermal conductivity of asphalt mixtures is 1.73 W·m^−1^·K^−1^ for AC 11 and 2.01 W·m^−1^·K^−1^ for AC 16. According to the transient method, the mean thermal conductivity values were 1.94 W·m^−1^·K^−1^ for AC 11 and 2.20 W·m^−1^·K^−1^ for AC 16. The range of obtained results is consistent with values reported in the literature [15]. In both methods, the AC 16 asphalt concrete showed higher thermal conductivity than the AC 11 concrete. To determine whether differences between the results for the two materials were statistically significant, an analysis of variance (ANOVA) was conducted. Levene’s and Tukey’s statistical tests were applied at a significance level of α = 0.05. After confirming the assumptions of the analysis, a statistically significant difference was found between the mean thermal conductivity values of the two tested asphalt mixtures. According to the authors, this difference may result from the amount of bitumen contained in the mixtures. The AC 11 mixture had a relatively high bitumen content of 5.8% by mass, while the AC 16 mixture contained only 4.8%. Considering the low thermal conductivity of bitumen, approximately 0.25 W·m^−1^·K^−1^ [13,14], the observed trend in the results is consistent with expectations.

C 16/20 cement concrete with dolomite aggregate shows higher thermal conductivity values than the C 35/45 concrete with granite aggregate. Using the ANOVA method described earlier, this difference was confirmed to be statistically significant. According to the authors, the primary factor influencing this difference was the type of aggregate. Cement concrete containing dolomite aggregate shows significantly higher thermal conductivity than concrete made with granite aggregate [20]. The results for cement concrete show greater variability compared to asphalt mixtures. In the steady-state method, the average coefficient of variation is approximately 2.2% for asphalt mixtures and 4.0% for cement concretes. In the transient method, it is around 3.0% and 5.4%, respectively. However, these values are not very high. Therefore, it was assumed that the obtained thermal conductivity values for the tested materials were determined correctly.

### 3.2. Comparison of the Test Results Obtained by Two Measurement Methods

**Figure 7:**
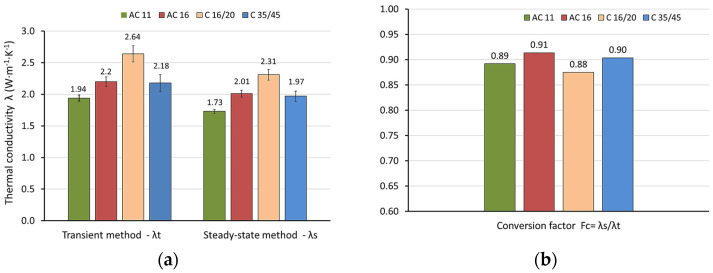
Thermal conductivity: (**a**) dependence on the type of tested material and test method; (**b**) conversion factor of results obtained by the transient method to the steady-state method.

The next stage of the analysis included a comparison of the test results obtained using the two methods. The mean values of the thermal conductivity test results of all materials using the steady-state and transient methods, together with the coefficients of variation (%), are presented in Table 12. The percentage difference in the mean thermal conductivity values in relation to the mean value obtained using the transient method is also shown. The test results are also presented graphically in Figure 7.

Based on the obtained mean thermal conductivity results, the conversion factor $F_{C}=\lambda_{S}\cdot \lambda_{t}^{-1}$ was determined. This factor allows the conversion of measurement results obtained using the transient method to values obtained using the steady-state standard method. The conversion factor $F_{C}$ can be used to quickly estimate the thermal conductivity value for road materials based on faster and cheaper measurements using the non-standard transient method. The conversion factor values are shown in Figure 7.

In all cases studied, the transient method shows higher thermal conductivity values compared to the steady-state method. This tendency is consistent with the literature [23]. For the AC 11 asphalt mixture, the difference is 10.8%, while for AC 16 it is 8.6%. The average difference in the results obtained by the two methods for both asphalt mixtures is 9.7%. For cement concretes, slightly larger differences are observed. It is 12.5% for C 16/20 and 9.6% for C 35/45, respectively. The average value for both cement concretes is approximately 11.1%. Using ANOVA, statistically significant differences in these results are confirmed (α = 0.05). The transient method shows greater variability in the results compared to the steady-state method. Taking into account all the variants analyzed, the transient method shows an average coefficient of variation of 4.2%, and the steady-state method of 3.1%. For all tested road materials, the mean conversion factor ($\bar{F_{C}}$) of 0.9 was obtained. This value can be used as a multiplier for the transient measurement results. This approach enables the estimation of thermal conductivity values for road materials that may be difficult to obtain using the standard steady-state method. However, it should be noted that the proposed conversion factor value was determined under assumed test conditions. Its broader applicability requires verification through further laboratory testing.

When analyzing the above results, it is important to remember the advantages and limitations of the measurement methods. The transient method enables quicker and easier measurements compared to the steady-state method, but it lacks standardized procedures for testing various materials. In contrast, the steady-state method, due to its isolated test system, yields results with lower variability. However, the steady-state method involves higher financial costs related to the purchase of more complex equipment. Additionally, it is more difficult and time-consuming to perform, especially when testing large and heavy specimens of non-homogeneous materials, such as asphalt mixtures and cement concretes. Therefore, it is essential to remain aware of the differences in thermal conductivity values obtained using these two methods when evaluating road pavement materials.

Further work is expected to expand the scope of research to include other road materials. In response to the increasing need for environmental protection, the planned investigations will focus on mixtures containing recycled materials as well as cold asphalt mixtures.

## 4. Conclusions

The results of the conducted research allow for the formulation of the following conclusions:

- Both the steady-state and transient methods are suitable for measuring the thermal conductivity of road materials. For the tested specimens, the steady-state method provides thermal conductivity values ranging from 1.73 to 2.31 W·m^−1^·K^−1^, while the transient method yields higher values, ranging from 1.94 to 2.64 W·m^−1^·K^−1^.
- Statistically significant differences (α = 0.05) were observed between the results obtained using the two methods. The average difference is estimated at 9.7% and 11.1% for asphalt mixtures and cement concretes, respectively.
- Estimating and confirming a statistically significant difference between the test results obtained using the two measurement methods is essential. When performing thermal analysis of road pavement structures, careful consideration should be given to the method used to determine thermal conductivity, as it is a fundamental material parameter. This is particularly important in areas affected by the UHI effect and for road sections used as heat collectors.
- The results of measurements obtained using the transient method show higher variability. The average coefficient of variation is estimated at 4.2% and 3.1% for the transient method and the steady-state method, respectively.
- According to the authors, the transient method can be widely applied due to its simplicity and practicality, especially when testing large and heavy road material specimens. However, a correction should be applied to account for the differences between the results obtained using the standardized steady-state method and the transient method.
- The proposed conversion factor ($\bar{F_{C}}$ = 0.9) can be used to quickly estimate the thermal conductivity values of road materials based on faster and more cost-effective measurements obtained using a non-standardized transient method.

## Figures and Tables

**Table 1:** Comparison of the two measurement methods.

Steady-State Method	Transient Method
Specified standard procedure for laboratory tests	No standard procedure. Tests performed according to the guidelines of the measuring device manufacturers
Method used in laboratory conditions	Method can be used both in laboratory and field conditions
More expensive testing equipment	Cheaper testing equipment
Difficult testing of large and heavy specimens	Relatively easy testing of large and heavy specimens
Possibility of testing specimens of limited dimensions	Possibility of testing specimens and elements with different dimensions and geometries
Longer measurement time for a single specimen	Shorter measurement time for a single specimen
Lower variability of results	Higher variability of results

**Table 2:** Specification of aggregate gradation.

Sieve Size (mm)	AC 11 Mass Passing (%)	AC 16 Mass Passing (%)
22.4	—	100
16	100	98
11.2	97	78
8	80	63
2	48	38
0.125	10	7
0.063	9	6

**Table 3:** Components of asphalt mixtures.

Material	Type	Apparent Density (g·cm^−3^)	Amount in AC 11 (%)	Amount in AC 16 (%)
aggregate 8/16	limestone	2.71	—	39.0
aggregate 8/12	dolomite	2.81	18.8	—
aggregate 2/8	limestone	2.72	—	17.1
aggregate 2/8	dolomite	2.81	24.5	—
aggregate 0/4	limestone	2.71	33.0	18.1
aggregate 0/2	sandstone	2.66	14.1	18.1
mineral filler	limestone	2.70	3.8	2.9
road bitumen	50/70	1.023	5.78	4.78
agent	adhesion	—	0.02	0.02

**Table 4:** Properties of asphalt mixtures.

Parameter	Standard	Results AC 11	Requirements AC 11 [33]	Results AC 16	Requirements AC 16 [33]
Density (g·cm^−3^)	EN 12697-5 [34]	2.478	—	2.503	—
Bulk density (g·cm^−3^)	EN 12697-6 [35]	2.432	—	2.395	—
Void ratio VV (%)	EN 12697-8 [36]	1.9	1.0–3.0	4.3	3.0–6.0
Voids filled with bitumen VFB (%)	EN 12697-8 [36]	87.9	75–93	72.3	60–80
Voids of mineral aggregate VMA (%)	EN 12697-8 [36]	15.7	min. 14	15.5	min. 14

**Table 5:** Specification of aggregate gradation.

Sieve Size (mm)	Mass Passing (%)
22.4	100
16	75
8	57
4	36
2	29
1	18
0.5	11
0.25	5

**Table 6:** List of specimens.

Material	Coarse Aggregate	Number of Specimens
AC 11	granite	8
AC 16	limestone	8
C 16/20	dolomite	8
C 35/45	granite	8

**Table 7:** Test results of thermal conductivity λ (W·m^−1^·K^−1^) using the steady-state method.

Specimen Number	AC 11	AC 16	C 16/20	C 35/45
1	1.75	2.01	2.30	2.03
2	1.72	1.95	2.19	1.92
3	1.71	2.02	2.25	1.98
4	1.79	1.99	2.26	1.86
5	1.73	2.09	2.33	2.05
6	1.74	1.93	2.27	1.92
7	1.68	2.07	2.41	2.10
8	1.75	2.02	2.45	1.90
**Mean value (W·m^−1^·K^−1^)**	**1.73**	**2.01**	**2.31**	**1.97**
**Standard deviation (W·m^−1^·K^−1^)**	**0.03**	**0.05**	**0.09**	**0.08**
**Coefficient of variation (%)**	**1.8**	**2.7**	**3.8**	**4.2**

**Table 8:** Test results of thermal conductivity λ (W·m^−1^·K^−1^) of the AC 11 asphalt mixture using the transient method.

Specimen Number	Series 1	Series 2	Series 3	Mean Value
1	1.95	1.94	1.99	1.96
2	1.94	1.86	1.88	1.90
3	2.01	2.03	1.98	2.00
4	1.93	1.93	1.88	1.91
5	1.92	1.92	1.91	1.92
6	1.90	1.91	1.84	1.88
7	2.07	2.01	1.96	2.01
8	1.91	1.99	1.91	1.93
		**Mean value (W·m^−1^·K^−1^)**		**1.94**
		**Standard deviation (W·m^−1^·K^−1^)**		**0.05**
		**Coefficient of variation (%)**		**2.5**

**Table 9:** Test results of thermal conductivity λ (W·m^−1^·K^−1^) of the AC 16 asphalt mixture using the transient method.

Specimen Number	Series 1	Series 2	Series 3	Mean Value
1	2.07	2.11	2.25	2.14
2	2.21	2.17	2.17	2.18
3	2.17	2.16	2.19	2.18
4	2.07	2.03	2.18	2.09
5	2.20	2.26	2.28	2.25
6	2.27	2.24	2.25	2.25
7	2.30	2.37	2.37	2.35
8	2.07	2.13	2.32	2.17
		**Mean value (W·m^−1^·K^−1^)**		**2.20**
		**Standard deviation (W·m^−1^·K^−1^)**		**0.08**
		**Coefficient of variation (%)**		**3.5**

**Table 10:** Test results of thermal conductivity λ (W·m^−1^·K^−1^) of the C 16/20 cement concrete using the transient method.

Specimen Number	Series 1	Series 2	Series 3	Mean Value
1	2.60	2.60	2.81	2.67
2	2.72	2.69	2.70	2.70
3	2.36	2.46	2.28	2.37
4	2.89	2.50	2.58	2.66
5	2.77	2.64	2.68	2.70
6	2.44	2.79	2.39	2.54
7	2.85	2.58	2.87	2.77
8	2.66	2.78	2.76	2.73
		**Mean value (W·m^−1^·K^−1^)**		**2.64**
		**Standard deviation (W·m^−1^·K^−1^)**		**0.13**
		**Coefficient of variation (%)**		**4.9**

**Table 11:** Test results of thermal conductivity λ (W·m^−1^·K^−1^) of the C 35/45 cement concrete using the transient method.

Specimen Number	Series 1	Series 2	Series 3	Mean Value
1	2.13	2.11	2.16	2.13
2	1.95	1.92	1.99	1.95
3	2.23	2.19	2.22	2.22
4	2.17	2.16	2.16	2.16
5	2.36	2.33	2.38	2.36
6	2.21	2.19	2.21	2.21
7	2.10	2.09	2.07	2.09
8	2.35	2.31	2.32	2.33
		**Mean value (W·m^−1^·K^−1^)**		**2.18**
		**Standard deviation (W·m^−1^·K^−1^)**		**0.13**
		**Coefficient of variation (%)**		**6.0**

**Table 12:** Summary of thermal conductivity test results using two measurement methods.

Material	Type of Aggregate	Mean Value $\lambda_{t}$ (W·m^−1^·K^−1^) Transient Method	Coefficient of Variation (%)	Mean Value $\lambda_{S}$ (W·m^−1^·K^−1^) Steady-State Method	Coefficient of Variation (%)	Difference in Mean Values (%) ${(\lambda }_{t}-\lambda_{S})\cdot \lambda_{t}^{-1}$
AC 11	dolomite	1.94	2.5	1.73	1.8	10.8
AC 16	limestone	2.20	3.5	2.01	2.7	8.6
C 16/20	dolomite	2.64	4.9	2.31	3.8	12.5
C 35/45	granite	2.18	6.0	1.97	4.2	9.6

## Data Availability

The original contributions presented in the study are included in the article. Further inquiries can be directed to the corresponding author.
